# Superhydrophobic Nanocoatings as Intervention against Biofilm-Associated Bacterial Infections

**DOI:** 10.3390/nano11041046

**Published:** 2021-04-19

**Authors:** Yinghan Chan, Xun Hui Wu, Buong Woei Chieng, Nor Azowa Ibrahim, Yoon Yee Then

**Affiliations:** 1 Department of Life Sciences, School of Pharmacy, International Medical University (IMU), Bukit Jalil, Kuala Lumpur 57000, Malaysia; yinghan.chan@gmail.com; 2 School of Postgraduate Studies, International Medical University (IMU), Bukit Jalil, Kuala Lumpur 57000, Malaysia; XunHui.Wu@outlook.com; 3 Department of Chemistry, Faculty of Science, Universiti Putra Malaysia (UPM), Serdang 43400, Malaysia; chieng891@gmail.com (B.W.C.); norazowa@upm.edu.my (N.A.I.); 4 Department of Pharmaceutical Chemistry, School of Pharmacy, International Medical University (IMU), Bukit Jalil, Kuala Lumpur 57000, Malaysia

**Keywords:** biofilm, polymicrobial infections, nosocomial infections, superhydrophobic, nanomaterials, anti-biofilm surfaces

## Abstract

Biofilm formation represents a significant cause of concern as it has been associated with increased morbidity and mortality, thereby imposing a huge burden on public healthcare system throughout the world. As biofilms are usually resistant to various conventional antimicrobial interventions, they may result in severe and persistent infections, which necessitates the development of novel therapeutic strategies to combat biofilm-based infections. Physicochemical modification of the biomaterials utilized in medical devices to mitigate initial microbial attachment has been proposed as a promising strategy in combating polymicrobial infections, as the adhesion of microorganisms is typically the first step for the formation of biofilms. For instance, superhydrophobic surfaces have been shown to possess substantial anti-biofilm properties attributed to the presence of nanostructures. In this article, we provide an insight into the mechanisms underlying biofilm formation and their composition, as well as the applications of nanomaterials as superhydrophobic nanocoatings for the development of novel anti-biofilm therapies.

## 1. Introduction

Polymicrobial infections are widely regarded as the most common causes for inflammation in surrounding tissues and the failure of implanted biomaterials. These pathogenic microbials can proliferate rapidly, resulting in nosocomial infections that constitute a major public health crisis as they can lead to extended hospital stay, long-term disabilities, increased socioeconomic burden, as well as increased morbidities and mortalities [1,2]. An estimate by the World Health Organization (WHO) revealed that approximately 15% of hospitalized patients suffer from nosocomial infections, where the prevalent types of infection include catheter-associated urinary tract infections, central line-associated bloodstream infections, ventilator-associated pneumonia, as well as surgical site infections [2]. Most microorganisms can develop various types of survival mechanisms in adapting to their surrounding environment, which allow them to sustain activity against host immune responses and antimicrobial therapeutics. Over the years, studies have established that bacterial populations generally attach to solid substrates for their survival, forming dense microbial communities known as biofilms, whereby these bacteria are embedded in extracellular polymeric matrix that can be composed of polysaccharides, proteins, DNA, and water [1,3]. Besides, the sustained survival of microbials within biofilms can also be attributed to altered metabolic activities, genetic adaptation, as well as modulated communications of microorganisms within the communities in microbial biofilms [4]. Thus, biofilm formation represents a remarkable virulence mechanism in the pathogenesis of multiple chronic bacterial infections, including those arising from *Staphylococcus aureus*, *Pseudomonas aeruginosa*, as well as *Escherichia coli*. Reports have also shown that approximately 80% of human microbial infections are the direct results of biofilm formation [5]. 

Although recent medical advancements have led to the development of various antimicrobial agents for the treatment of bacterial infections, pathogenic biofilms are often resistant to these agents. This may be attributed to the presence of genes for multidrug resistance, as biofilms are an ideal platform for the exchange of plasmids between communities of microbial cells. Notably, such recalcitrant properties of biofilms are found to be several orders of magnitude higher than those of planktonic bacteria, making them extremely challenging to be eradicated successfully [5,6,7]. Considering the number of individuals suffering from biofilm-associated device-related infections, the identification of novel therapeutic strategies targeting bacterial biofilms is of utmost importance to prevent and combat various infections resulting from the formation of bacterial biofilms. Moreover, the rapid development of implantable biomedical devices has brought the issue of medical device-associated infections to the forefront, as these implantable and prosthetic devices can easily become contaminated [8]. Over the years, various solutions have been proposed to mitigate the formation of biofilms. One strategy is by the inhibition of the initial attachment of microbials to biofilm-forming surfaces through alteration of the physicochemical properties of biomaterials. This is due to factors including surface charge, surface hydrophilicity, and biomaterial composition contributing to the rapidly increasing rate of medical device-associated infections [7,8,9]. Hence, superhydrophobic surfaces have gained increasing attention for this purpose due to their extremely low wetting properties, which can potentially reduce the colonization and adhesion of microbials, as well as preventing subsequent biofilm formation [7,9]. Superhydrophobic surfaces can be fabricated using various nanomaterials, such as polymer nanocomposites, carbon nanotubes, as well as metal nanoparticles [10]. In this review, we provide an insight into the basic composition of biofilms and the processes leading to biofilm formation. The potential of superhydrophobic surfaces as a novel anti-biofilm approach superior to those of conventional therapeutics and the advantages of nanomaterials in fabricating superhydrophobic surfaces will also be discussed, justified by various studies that are conducted in this field of research.

## 2. Bacterial Biofilms

Bacterial biofilm is a group of cooperative and coordinated unicellular microbes which are associated with physiological and structural complexity, and they are analogous to multicellular microorganisms. Bacterial biofilms can exist on a vast range of biotic surfaces, such as the skin, connective tissues, bones, airways, vascular endothelium, and intestinal mucosa, resulting in multiple types of tissue-associated chronic infections. Biofilms can also be associated with the development of infections from indwelling medical devices, such as catheters, sutures, orthopaedic implants, heart valves, intrauterine devices, and vascular grafts. Some examples of the biofilm pathogens that commonly result in medical device-associated bacterial infections are *Staphylococcus aureus*, *Staphylococcus epidermidis*, *Pseudomonas aeruginosa*, *Escherichia coli*, and *Klebsiella pneumoniae* (Table 1) [4,11,12]. Specifically, the formation of biofilms on medical devices used within the healthcare setting enables pathogens to persist as reservoirs which can be easily spread in patients [13]. The formation of bacterial biofilms can be attributed to the alternative dynamic and multifaceted lifestyle of microbials cells within the biofilm that confers them remarkable capability to survive in diverse environmental niches [13,14]. Generally, a diverse array of microbial cells can form biofilm that often consists of various species under normal conditions, whereby the maintenance of such a biofilm is regulated by intra- and intercellular communications via autoinducers [15]. During the process of biofilm formation, the microbes which are present in the host establish contact with a surface as part of a probabilistic process, driven by hydrodynamic force, gravitational force, as well as Brownian movement. Such surface attachment of microbials can either be reversible or irreversible, which is often dependent on the surrounding biological environment. The adhered microbial cells then proceed to penetrate host tissue for deriving nutrients and to prepare themselves for cellular division, forming a bacterial biofilm [14,16]. In the following sections, we discuss the basic structure and composition of a bacterial biofilm and provide a general overview on the steps and mechanisms leading to biofilm formation. 

### 2.1. Structure and Composition of Bacterial Biofilm

**Figure 1:**
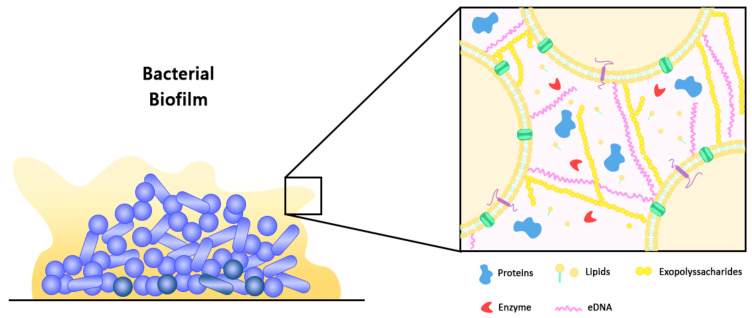
Schematic representation of a bacterial biofilm and its extracellular polymeric substance (EPS). Microcolonies of a mature biofilm are typically characterized by the presence of an EPS matrix that is composed of exopolysaccharides, proteins, extracellular DNA (eDNA), lipids, and enzymes. The EPS matrix acts as a protective barrier to shield the microbial community from external threats, including those of host defense mechanisms and antimicrobial therapeutics [12].

Biofilm can be defined as structured communities of microorganisms that are adherent to a biotic or abiotic surface, which are embedded in a matrix of self-producing extracellular polymeric substance (EPS). Typically, approximately 35% of the biofilm volume is made up of microorganisms, while the remaining volume is constituted by EPS [18]. As such, the production of EPS is the hallmark of biofilm formation, in which the EPS facilitates the attachment of microbial cells to surfaces and promotes cell-to-cell adhesion and aggregation [4,12]. At the same time, the matrix of EPS functions as a three-dimensional protective barrier that shields the microbial cells against external threats, which may include the host defence mechanisms and antimicrobial therapeutics. In addition, through the modulation of chemical and nutrient gradients, the EPS matrix can lead to the formation of a harsh biological environment that is essential for major virulence attributes [12]. Nevertheless, the function of EPS within the biofilm is vast and it has a variable composition between different microbial species. For example, cellulose produced by *Escherichia coli* contributes to increased resistance of the microbial communities to desiccation, whereas BslA, a bacterial hydrophobin of *Bacillus subtilis*, forms a water-resistant coat over the microbial communities [19]. In general, water constitutes the major part which accounts for approximately 97% of the EPS, with the remaining constituents such as exopolysaccharides (1–2%), proteins (more than 2%), DNA and RNA molecules (less than 1%), as well as ions making up the composition of biofilm (Figure 1) [18,19]. Certain host derived components including platelets, fibrin, as well as immunoglobulins may also be present in biofilms within complex host environments [12]. In terms of its architecture, the layout of biofilm comprises two major components, namely an area of closely packed microbial cells lacking eminent pores, and water channels that act as a simple circulatory system for the efficient transport of nutrients [8,20]. As biofilm is polymorphic in nature, its structure can also be altered in response to changes in the amount of nutrients, in which microcolonies grow faster in the presence of high glucose concentration, leading to increased biofilm thickness. On the contrary, low glucose concentration leads to reduced biofilm biomass, thereby restoring its former structure. Hydrodynamic condition is another factor that could affect the structure of biofilm, for instance, bacterial microcolonies become round in a laminar flow environment, whereas in a turbulent flow environment, bacterial microcolonies extend in downstream, having various phenotypes [20].

#### 2.1.1. Exopolysaccharides

Exopolysaccharides are the scaffold of biofilms that function to cross-link bacterial cells together within the biofilm. It has been established that a single bacterial species can produce various types of exopolysaccharides in different stages of biofilm formation, which are distinct in terms of their roles and functions (Table 2) [21,22]. Typically, these exopolysaccharides are highly charged to facilitate the absorption of water and ions from the surrounding environment, such as magnesium and calcium cations, which helps to shield the microbial cells from desiccation and buffer in response to pH changes. Besides, exopolysaccharides can also protect the biofilm community against harmful conditions, including ultraviolet irradiation and antimicrobial treatment [14,21]. Alginates, cellulose, and poly-N-acetylglucosamine (PNAG) are the most common exopolysaccharides present within the EPS of bacterial biofilms.

Alginate is a polysaccharide that can be found in the cell walls of brown algae and in multiple bacteria belonging to the genera *Azotobacter* and *Pseudomonas*. In terms of structure, uronic acid residues including β-D-mannuronic acid, depicted as “M block”, and α-L-guluronic acid, which is its C5 epimer, depicted as “G block”, build up the skeleton of alginate polymer via 1,4-glycosidic bonds [23,24]. The most prominent characteristic of alginates is their capability to bind with divalent cations in an efficient and selective manner, which results in the formation of alginate hydrogels and cross-linked polymeric scaffolds [25]. Naturally, alginates are often present in heteropolymeric forms with varying numbers and lengths of M and G blocks, as well as acetylated and non-acetylated residues. Although all alginates can exhibit a certain extent of viscoelastic properties, such physiochemical properties of alginates can be modified by modulating their molecular mass and composition, particularly the M to G block ratio. For instance, the intrinsic flexibility of alginates is influenced by the frequency of constituting blocks in the decreasing order of MG block, MM block, and GG block. The mechanical properties of alginate gels are also enhanced when the length of G block and molecular weight are increased [23,25,26]. Moreover, it has been shown that the structure–activity relationship plays an essential role in altering the physiochemical properties of alginates. For example, the acetylation of alginates can impact their properties in terms of polymer conformation, water-binding capacity, chain expansion, viscoelasticity, as well as molecular mass, leading to higher water absorption due to improved interaction of alginate side chains with water molecules [23,26]. The protonation of carboxylate groups in the alginate structural backbone can also result in the formation of hydrogen bonds, thereby increasing the viscosity of alginate gel [26]. 

Cellulose is the most abundant sugar-based biopolymer that can be found in multiple plants, animals, as well as in bacterial cells such as *Escherichia coli* and *Salmonella* spp. Structurally, cellulose is a linear, unbranched homopolysaccharide that is made up of β-1,4-linked glucosyl residues [27]. Typically, cellulose can form hydrogels in the presence of water, which allow the retention of an incredible amount of water attributed to the high surface area and numerous hydrogen-bonding sites. Such gelling ability of cellulose provides mechanical strength and protection to biofilms formed by the species of bacteria producing this polymer [24,28]. Besides, its production facilitates the binding of bacteria to host epithelial cells and reduces host immune responses, which are essential in establishing a commensal relationship [14,29]. Cellulose has also been shown to be responsible for cell aggregation in the pellicle, thereby allowing the biofilm to float to culture surface where oxygen is readily present to the bacterial cells. Moreover, cellulose can also shield bacterial biofilms from the mutagenic effects of ultraviolet irradiation [29]. The genes responsible for producing cellulose have various names depending on the bacterial species, for example, *Acetobacter* cellulose synthase (*acs*), bacterial cellulose biosynthesis (*bcs*), and cellobiose (*cel*). These genes are organized as an operon, whereby an operon consists of two conserved genes and a few accessory genes. The first conserved gene, such as *bcsA, acsA,* or *celA*, functions to encode cellulose synthase, followed by the second conserved gene, such as *bcsB*, *acsB*, or *celB*, which functions to encode cyclic dimeric guanosine monophosphate (c-di-GMP) binding protein. Today, c-di-GMP has been recognized as the key signalling molecule that promotes the formation of bacterial biofilm via the stimulation of cellulose synthesis [27,29]. 

PNAG is another example of an exopolysaccharide commonly found in biofilms. PNAG was first isolated and characterized from *Staphylococcus* spp., in which it has been referred to as the intracellular adhesin of polysaccharides, and it was further discovered in *Escherichia coli* as well as other Gram-negative and Gram-positive organisms [24,30]. PNAG is a positively charged linear homoglycan which is made up of β-1,6-linked 2-amino-2-deoxy-D-glucopyranosyl residues with different extent of N-acetylation and O-succinylation [14]. The synthesis of PNAG is dependent on the proteins encoded by genes in the intercellular adhesion (*ica*) operon, which consists of a regulatory gene *icaR* and four other biosynthetic genes, namely *icaA*, *icaB*, *icaC*, and *icaD*. This has been affirmed in several studies where it was demonstrated that *ica*-deleted PNAG-deficient strains lack the ability to produce biofilms [31,32]. Nevertheless, *ica*-independent formation of biofilms has been reported in Methicillin-resistant *Staphylococcus aureus*, in which the formation of this biofilm phenotype is mainly mediated by cell surface adhesion proteins and extracellular DNA [33,34]. Within biofilms, PNAG forms a protective matrix around bacterial populations whilst interacting with eDNA, which further reinforces the structure of biofilm matrix. PNAG also mediates intercellular adhesion, thereby promoting the accumulation of bacterial cells. Hence, PNAG is regarded as a major structural determinant of biofilms contributing to sustained, persistent and nosocomial bacterial infections [24,35].

#### 2.1.2. Proteins

Proteinaceous components of the EPS can include cell surface adhesins, the protein subunits of bacterial appendages such as pili and flagella, secreted extracellular proteins, as well as proteins of outer membrane vesicles. Cell surface proteins, pili and flagella typically contribute to the initial attachment of microbial cells to surfaces, at the same time, they are also involved in the migration of microbial cells along the surfaces, leading to surface colonization [36]. An abundance of secreted extracellular proteins can be observed in the EPS and they contribute to the structure and physiology of EPS, proving themselves to be an indispensable functional component for the formation of bacterial biofilms [37]. For instance, exopolysaccharides binding protein like lectins can facilitate the linkage of bacterial cells to the exopolysaccharides, resulting in a stabilized EPS matrix network. Besides, the biofilm-associated protein (Bap) family of high molecular mass surface-associated proteins contribute as adhesins for the primary attachment of microbial cells to abiotic surfaces as well as intercellular adhesion [21]. Moreover, certain matrix proteins are found to possess enzymatic properties towards the components of EPS. Examples include glycosyl hydrolase dispersin B, which hydrolyses polysaccharides, DNases, which degrade extracellular nucleic acids, as well as proteases, which target matrix proteins. Collectively, these proteins contribute to the reorganization of the EPS and facilitate degradation and dispersal or bacterial biofilms [36,37]. 

The roles of proteins in EPS have been affirmed in various studies throughout the years. Zhang et al. employed quantitative proteomics in studying the matrix-associated proteins obtained from various phases of *Pseudomonas aeruginosa* biofilms. The study found that there were increased numbers of nutrient metabolism related proteins over the period of biofilm growth, suggesting that matrix-associated proteins contributed to the formation of an integral and well-regulated microenvironment for the microbial cells, leading to nutrient acquisition, stress resistance, as well as the development and stability of the biofilm [38]. Another study by Wu et al. had identified several proteins that are involved in the formation of non-typeable *Haemophilus influenzae* biofilm. For instance, glyceraldehyde-3-phosphate dehydrogenase, DnaK2, ornithine carbamoyltransferase, and 5′-nucleotidase are the surface-associated proteins involved during the early phase of biofilm formation, where they play a role in facilitating bacterial attachment and adhesion [39]. Similarly, Valle et al. investigated the roles of Bap in *Staphylococcus aureus* biofilm. It was shown that Bap promotes the adhesion of pathogens via glycoprotein 96 (Gp96) interaction whilst preventing the internalization of the pathogens into host epithelial cells. The findings suggested that Bap protein facilitates the formation of bacterial aggregates on surfaces and promotes immune evasion, thereby establishing long-term persistent biofilm infections [40]. Nonetheless, the functions of proteins during biofilm growth remained largely underexplored, necessitating further studies to clearly elucidate the underlying roles of proteins in biofilm formation [41]. 

#### 2.1.3. Extracellular DNA

Apart from exopolysaccharides and proteins, extracellular DNA (eDNA) has been recognized as another substantial component of biofilm EPS. The importance of eDNA in biofilm formation was first observed in *Pseudomonas aeruginosa*, whereby treatment with DNase affected the early stages of biofilm growth and resulted in nearly complete removal of microbial cells from the surface [42]. Today, it becomes prominent that many Gram-positive and Gram-negative bacterial cells can release eDNA within their biofilms, such as *Staphylococcus* spp., *Vibrio cholerae*, *Enterococcus faecalis*, and *Helicobacter pylori*, in which eDNA possesses various roles and functions essential for the growth of biofilms [43]. Generally, the physical characteristics of the eDNA molecule allow it to provide strong adhesion for microbial cells to attach on surfaces. However, studies found that eDNA has little significance on mature biofilms as its contribution to the stability of biofilms is only observed in the initial phases of biofilm growth, demonstrated by the disintegration of bacterial aggregates after DNase treatment or induced aggregate and subsequent biofilm formation upon the addition of DNA [43,44]. Such adhesion promoting property of eDNA further leads to increased mechanical strength of bacterial biofilms, shielding them from physical barriers such as shear stress [45]. Besides, eDNA promotes the efficient flow of microbial cells throughout the furrow network via the maintenance of coherent cell alignments, which subsequently avoids congestion and allows efficient supply of microbial cells to the migrating front, a process which is crucial for the initiation of microcolony formation in biofilms [43]. The presence of eDNA in bacterial biofilms is also commonly associated with the secretion of bacterial nucleases, making it a flexible and shapeable structural component that can be adjusted according to the requirements of the biofilm bacterial population [46]. Moreover, eDNA can confer protection to biofilm microbial communities against antibiotics and the host immune system. This is attributed to the ability of eDNA to bind cations, which enables the direct chelation of cationic antimicrobial peptides produced by the host immune system. eDNA also contributes to the increased pathogenicity of biofilms through the upregulation of genes involved in antimicrobial resistance [42,45]. Despite most research indicating the functional relevance of eDNA to the growth and rigidity of bacterial biofilms, a study by Wang et al. has reported that eDNA destabilized and hindered the development of *Salmonella enterica* biofilm on abiotic surfaces. This is deduced from the findings that remarkably more biofilms were formed in the presence of DNase, whereas biofilm formation decreased upon the addition of eDNA [47]. Hence, detailed identification of the interactions between eDNA and bacterial biofilms will be a future research task to pave the way for the development of eDNA-targeted biofilm control strategies.

### 2.2. Formation of Bacterial Biofilm

**Figure 2:**
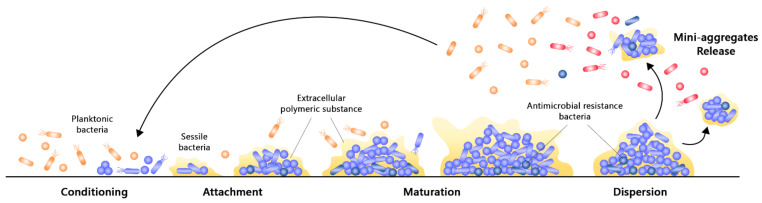
Schematic representation of the different stages in biofilm formation. The process begins with a reversible attachment of planktonic bacterial cells and their adhesion to the surface. Once they form a monolayer, the bacterial cells become irreversibly attached and the production of extracellular polymeric substance will be initiated. As the biofilm matures, microcolonies are formed where bacterial cells aggregate and accumulate in multiple layers. Finally, the mature biofilm disperses and releases planktonic bacterial cells, starting a new cycle of biofilm formation.

The formation of biofilm is a complex, dynamic, multistep, and cyclic process involving multiple bacterial species. Their formation typically results from a default defence mechanism of the microbial cells to achieve a favourable habitat and retain nutrients, which essentially ensures survival in extreme environments [8,15]. It is an outcome of physical, chemical, and biological events that is mediated by a special type of signalling known as quorum sensing (QS). QS is an intracellular signalling mechanism responsible for the regulation of gene expressions in response to small diffusible signalling molecules known as autoinducers [48]. As these autoinducers are constantly produced by the bacterial cells, their level is directly proportional to the cell density. Upon reaching the quorum level, which is a critical threshold concentration of autoinducers at a specific cell density, the binding of autoinducer receptor can result in the activation or repression of various target genes. Such modulation of the QS process enables bacterial cells to present a unified response via the maintenance of optimal biofilm size and coordination of virulence phenotypes [5,49]. In short, QS modulates the expressions of multiple genes that are involved in the control of motility, the synthesis of biosurfactant, and the production of EPS, in which all of them are necessary for the formation of biofilm and effective stress response towards harsh environmental conditions [15,49,50]. Although the QS mechanism in biofilm formation is generally similar amongst the various species of bacteria, slight differences may be present between them. Most of the Gram-negative bacteria such as *Pseudomonas aeruginosa* utilize N-acyl homoserine lactones (AHLs) as the autoinducers, while some Gram-negative organisms such as *Vibrio cholera* utilize autoinducer 2 (AI-2) as their signalling molecule. On the contrary, AHLs are not found to be produced by any of the Gram-positive bacteria; instead, organisms such as *Staphylococcus aureus* and *Streptococcus* spp. utilize small post-translationally processed peptide signal molecules known as autoinducing peptides (AIPs) as their autoinducers for QS [48,51]. The process of biofilm formation can be mainly divided into three different stages, namely the initial attachment, followed by the maturation of microcolonies, and lastly dispersion (Figure 2). These can be affected by changes within the biological environment, such as hydrodynamics and availability of nutrients, and it is highly dependent upon the physical properties of the surface, such as roughness and hydrophilicity [8,18]. 

Conditioning and attachment of planktonic bacterial cells to a surface are initial steps of the biofilm formation. As most pathogenic bacterial cells are opportunistic, physical forces including electrostatic interactions and van der Waal’s forces can facilitate the attachment of planktonic microbial cells to surfaces. Chemotaxis refers to the directed movement of microbial cells towards a nutrient source along the concentration gradient in mobile fluids, in which it promotes the adhesion of microbial cells on surfaces via cell–surface interactions [8,52]. Bacterial appendages such as fimbriae, flagella or pili can also provide strength to the interaction between bacterial cells and the attached surface [18]. This forms a conditioning layer on surfaces, which is a reversible attachment due to the weak and transient interactions between the bacterial cells and surface. This layer consists of proteins such as fibrinogen, collagen, fibronectin, vitronectin, and laminin, as well as von Willebrand factor and polysaccharides [12,52]. Nevertheless, if the attractive forces are favoured over repulsion, certain irreversibly attached cells may become immobilized and irreversibly attached, followed by the formation of a monolayer with strong adhesive properties [52,53]. Once the attachment of microorganisms to a surface becomes stable, chemical signalling within the EPS triggers the multiplication and division of microbial cells, which results in the formation of microcolonies. Within the microcolonies, processes including substrate exchange, the distribution of metabolic products, and the excretion of metabolic end products occur between microbial cells via a coordinated response [54].

During the maturation phase, the adhered microbial cells communicate with one another through the production of autoinducer signals, which facilitates QS and corresponds to signalling cues that aid in virulence and gene regulation [55]. In this phase, microbial cells begin the secretion of EPS, which encloses them and stabilizes the biofilm network. Overall, two stages of maturation have been observed. Stage 1 maturation primarily involves cell-to-cell communication, whereas stage 2 maturation is characterized by the increasing size and thickness of the microcolony which forms a microcolony [8]. Interstitial voids will also be formed within the EPS, in which these are water channels that function to distribute essential nutrients and eliminate waste products from the biofilm bacterial communities. Towards the end of biofilm maturation, a structured multicellular community will be formed. These microorganisms are typically protected against external threats and have altered gene expression, which induces the production of virulence factors and enhances their survival [12,56].

Finally, dispersion is an important stage in the late processes of biofilm formation as the microbial cells within the biofilm undergo rapid multiplication and detachment, as well as the switching from sessile into motile form. This results in the expansion of bacteria from one region to another, thereby spreading infection [54,57]. Biofilm dispersion is a process mediated by levels of oxygen and nutrients. As biofilm matures, the availability of resources decreases whilst toxic products increase. Therefore, the dispersion of microbial cells to other regions of the host body or medical implants as either single or as clumps helps them to expand, obtain nutrition, as well as to eliminate stress-inducing conditions and accumulated waste [58]. Besides, the process of detachment is also mediated by various saccharolytic enzymes that are produced by the microbial communities within the biofilm. For example, alginate lyase produced by *Pseudomonas* spp. and N-acetyl-heparosan lyase produced by *Escherichia coli* can lead to the efficient lysis of the EPS matrix and the eventual detachment of microbial cells from surfaces [18]. Lastly, upon microbial release, they can either develop more biofilms or present as planktonic cells that freely float on the surface through the upregulation of flagella proteins, which aids in their motility [54].

## 3. Superhydrophobic Surfaces

**Figure 3:**
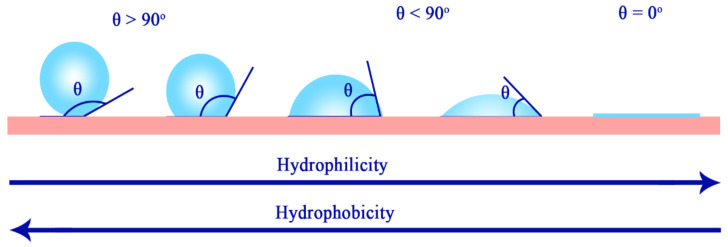
Schematic representation of the contact angle (θ) of a water droplet with a solid surface. The contact angle is typically used as a measurement of the wettability index of a surface, in which a contact angle of 0° indicates superhydrophilicity, less than 90° indicates hydrophilicity, greater than 90° indicates hydrophobicity, and greater than 150° indicates superhydrophobicity.

**Figure 4:**
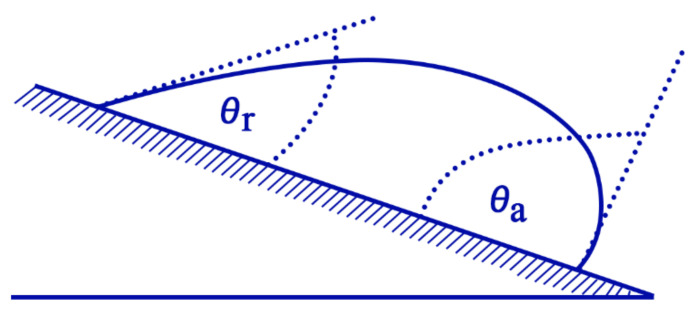
Schematic representation of advancing (θ_a_) and receding (θ_r_) contact angles on a tilted surface. Advancing contact angle can be referred to as a measure of solid–liquid cohesion, whereas receding contact angle can be referred to as a measure of solid–liquid adhesion. The difference between these angles is known as contact angle hysteresis, whereby a greater contact angle hysteresis indicates greater stickiness of the surface.

Surface wettability refers to an interface phenomenon between a solid support and a liquid, whereby the behaviour of the liquid is regarded as the indicator for wettability. Depending on how a water droplet interacts with a solid surface, the surface can be classified as either hydrophilic, hydrophobic, or superhydrophobic (Figure 3) [59,60]. A superhydrophobic surface can maintain air at the solid–liquid interface upon contact with water. It can be defined as a surface which exhibits a high apparent contact angle of more than 150°, low contact angle hysteresis of less than 10°, and a sliding angle of less than 10° [59]. Contact angle refers to the angle where the liquid–air interface meets the solid–liquid interface, and it is commonly used to determine the wettability of a flat surface. Contact angle hysteresis refers to the difference between advancing and receding contact angles formulated by a water droplet on an inclined surface, and it is commonly utilized to determine the repellent characteristic of a solid surface towards water droplets (Figure 4) [61,62]. As larger hysteresis indicates greater stickiness of the surface, low hysteresis is desired in superhydrophobic surfaces as it allows water droplets to roll off from the surface easily [61]. Such a phenomenon is known as the Lotus effect, whereas if a surface simultaneously displays high contact angle with large contact angle hysteresis, it is known as the rose petal effect [63,64]. 

**Figure 5:**
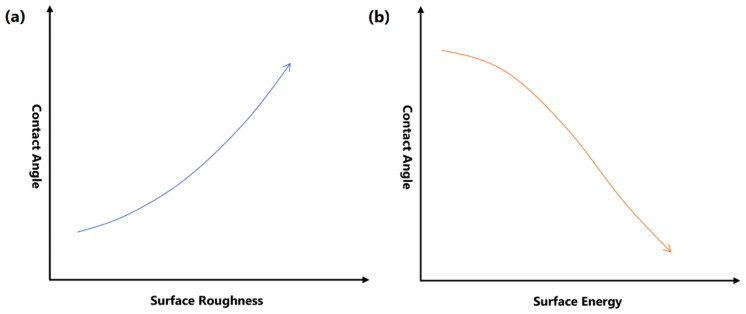
The correlation between surface roughness and surface energy with water contact angle: (**a**) Higher surface roughness leads to higher contact angle due to presence of air bubbles within surface protrusions which reduces contact area and frictional drag of water droplets with the surface; (**b**) Lower surface energy leads to higher contact angle due to poor adherence of water droplets to the surface.

Surface free energy and the roughness of the surface are the primary factors involved in the control of surface wettability (Figure 5). Superhydrophobic surfaces can be commonly achieved by lowering the surface energy. When the surface energy is low, molecules of the water droplet can be strongly attracted to each other instead of adhering to the surface, thereby resulting in higher contact angle, which suggests that water droplets can be easily repelled from the surface [65]. An uneven, rough surface is also crucial in achieving an optimal superhydrophobic property. In such surfaces, water droplets do not have contact with all surface points due to the presence of air bubbles that are caught within the ups and downs of the surface. Along with low surface energy, the presence of air pockets prevents the penetration of water droplets into the valley, leading to reduced contact area and decreased frictional drag. Subsequently, water droplets can flow off the surface easily and effectively [66].

**Figure 6:**
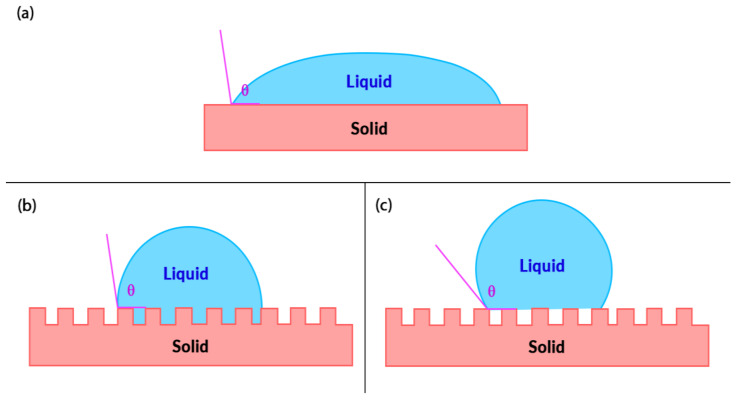
Schematic representation of different wetting models: (**a**) Young’s model, which describes the basic wetting phenomenon on an ideal, homogenous surface; (**b**) Wenzel’s model, which describes wetting phenomenon that considers surface roughness; (**c**) Cassie–Baxter’s model, which is a more complex model that describes wetting phenomenon on a heterogenous surface.

Over the years, the wettability of solid surfaces and their solid–liquid physical interactions have been explained through various wetting models (Figure 6). The first wetting model is the Young’s model, which states that the contact angle of a surface is a measure of the surface energy equilibrium of the solid–vapour, solid–liquid, and liquid–vapour interfaces. However, Young’s wetting model is only applicable to smooth and homogenous surfaces that are inert to the fluid they encounter [59,67]. This led to the elucidation of Wenzel and Cassie–Baxter models that correlated surface roughness and its wettability. In Wenzel’s model, the liquid droplet completely fills and permeates the protrusions of a rough solid surface upon contact, forming a “fully-wetted” interface. Low surface tension liquids tend to present very low contact angles in Wenzel’s state [60,68]. Wenzel also proposed that with increasing surface roughness, a hydrophilic surface will become more hydrophilic, whilst a hydrophobic surface becomes more hydrophobic [60]. On the contrary, the liquid droplet does not completely wet the surface in the Cassie–Baxter model. Instead, air pockets are trapped underneath the liquid droplet leading to an increased water contact angle, thereby allowing effective fluid flow [68]. 

Nevertheless, the stability of the Cassie–Baxter state is mostly dependant on applied conditions such as hydraulic pressure, vibration, or changes in surface energy, as the escape of air pockets can force its transition into the Wenzel state of wetting [69]. Pure Wenzel or Cassie–Baxter wetting states are not commonly observed in nature, as both states can often coexist on a micro or nanotextured hydrophobic substrate. As such, the Cassie–Baxter regime of air trapping is known to be metastable due to the possibility of irreversible transition towards another regime [70,71]. Although the contact angles in both states are comparable, the hysteresis will be significantly altered during the shifting of wetting state, in which it is found to be approximately 10 to 20 times larger in the Wenzel state. This observation is of practical importance as increased hysteresis indicates better adherence of water droplets to the surface, which is a clear distinction from the expected phenomenon in a superhydrophobic state. Moreover, the shifting of wetting states from Cassie–Baxter to Wenzel regime also suppresses the self-cleaning effect whilst increasing its friction properties [71]. Hence, the maintenance of air pocket entrapment is crucial for the Cassie–Baxter state in generating an ideal superhydrophobic surface. Thermodynamic equilibrium should be maintained to ensure the stability of the Cassie–Baxter regime, as vapour phase invasions such as dew condensation or drop evaporation can affect the water-repellent property of a surface [71,72]. 

## 4. Nanomaterials for Fabrication of Superhydrophobic Surfaces

Throughout the years, efforts have been made to fabricate superhydrophobic surfaces inspired by those of natural plants and animals. Various natural materials such as lotus leaf, animal species, as well as their specific parts have been found to possess substantial superhydrophobic properties (Table 3) [73,74,75,76,77]. Lotus leaf is the most popular example of a naturally occurring superhydrophobic surface. Its superhydrophobic property is thought to be attributed to the dense layer of epicuticular waxes and microscopic bumps present on the leaf surface. The hydrophobic nature of epicuticular waxes allows water droplets to remain high enough, which subsequently reduces contact area and increases contact angle to be larger than 90°. Moreover, the microscopic bumps enable air to be trapped within the bump spaces, resulting in a contact angle of larger than 150°. As a result, water droplets become nearly spherical and they can roll off the leaves smoothly and effectively. At the same time, dirt from the surface on the leaves can be collected and removed, demonstrating a self-cleaning effect [76,78]. 

The fabrication of superhydrophobic surfaces involves two major factors, namely, low surface energy and optimum roughness in the micro- or nano range. Generally, artificial superhydrophobic surfaces can be fabricated through various techniques, one of which is superhydrophobic nanocoating, which is an indirect surface modification technique that involves the creation of a new protective and multifunctional layer on the substrate with totally different properties. Unlike etching and lithography methods that directly modify the surface, superhydrophobic nanocoatings are not restricted by the range of substrates that can be used [79]. Nanotechnology is a field of scientific research concerning the know-how of the manufacturing and the understanding of the property, structure, and behaviour of nanomaterials, which are materials of nanoscale dimensions. Nanomaterials have been widely employed in the biomedical field for various applications, such as drug and gene delivery, biosensing, bioimaging, and tissue engineering [80,81]. With the advancement in the field of nanotechnology, functional superhydrophobic surfaces are becoming more facile and powerful due to the development of various nanomaterials that can be used to impart surface superhydrophobization with remarkable effects. As such, superhydrophobic nanocoating can be defined as a kind of superhydrophobic coating involving at least one nanoscaled raw material with a crucial role in depicting the coating’s properties, or the morphology of the superhydrophobic coating is in the nanoscale of at least one dimension [79,82]. Currently, three categories of nanomaterials are employed for the fabrication of superhydrophobic nanocoatings, namely, inorganic, organic, or inorganic–organic hybrid. These nanomaterials have gained increasing attention due to their unique and favourable features, such as biocompatibility, good processability, high flexibility, facile preparation, non-toxicity, and low cost [82,83]. There have been numerous studies performed with regard to the use of nanomaterials in the fabrication of superhydrophobic nanocoatings. In the following sections, we provide an overview of some of the work that had presented promising results in this field of research, in which the findings from these studies can provide new and interesting avenues for the future development of superhydrophobic surfaces.

### 4.1. Inorganic Nanomaterials

#### 4.1.1. Carbon-Based

**Figure 7:**
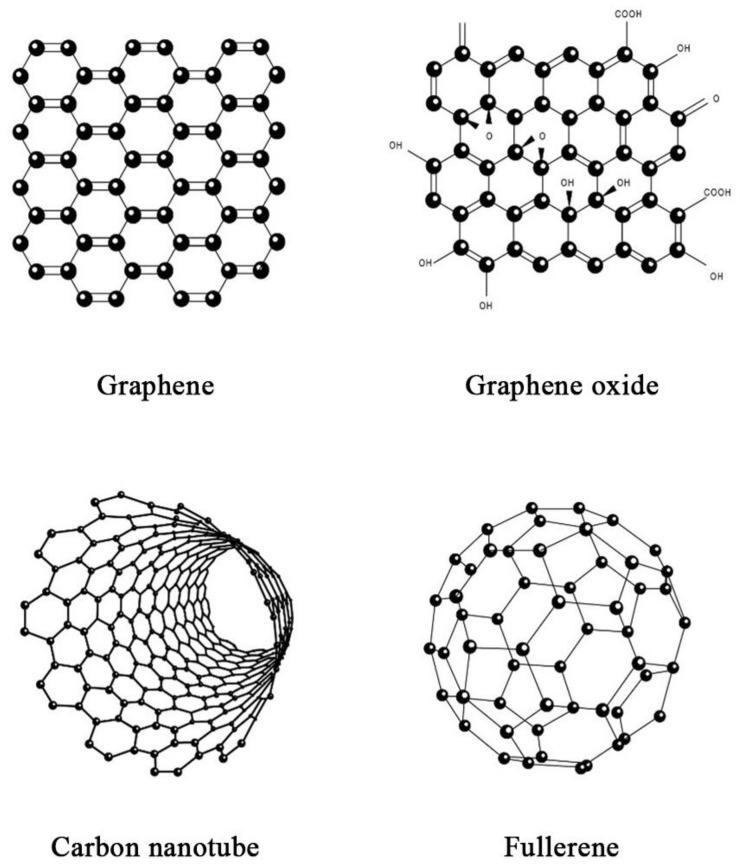
Structures of carbon-based nanomaterials.

The discovery of novel carbon materials, such as carbon nanotube (CNT), fullerene and graphene, has allowed carbon-based nanomaterials to become the focus of many fields, including the field of superhydrophobic nanocoating (Figure 7). This is attributed to the intrinsic stability of carbon, which is beneficial in maintaining the mechanical and chemical stabilities as well as the robustness of carbon-based nanomaterials [79,84].

Graphene is a nanomaterial that has emerged as one of the most promising materials in various applications due to its exceptional strength as well as thermal, mechanical, and optical properties. It is a thin sheet of mono-atomic carbon arranged in a crystalline lattice with a hexagonal honeycomb-like structure. The intrinsic material characteristics of graphene allow it to be constructed as micro- and nanostructures for superhydrophobic nanocoating applications [85]. In a study, Abbas et al. fabricated a stable self-cleaning superhydrophobic surface on copper alloy substrates using fluorinated graphene through a drop coating process. The superhydrophobic film presented with an irregularly stacked multi-layered microstructure. The remarkable superhydrophobicity of the fluorinated graphene films was proven by a high water contact angle of 167° and a minimal water sliding angle of less than 4°. Additionally, a corrosion test revealed that the superhydrophobic surface was resistant to high abrasive forces, demonstrated by a negligible drop in the water contact angle to 166° [86]. A similar study by Wang et al. had fabricated superhydrophobic film on a copper surface using reduced graphene sheets via electrophoretic deposition. The superhydrophobicity of the film was demonstrated by a water contact angle of 150.4°. This phenomenon can be attributed to the presence of trapped air in the island-like structure of the reduced graphene sheet, thereby reducing the contact surface area [87]. Apart from pristine graphene, graphene oxide has also been widely employed for the fabrication of superhydrophobic surfaces. It is a derivative of graphene with the presence of oxygen functional groups, which can be prepared through the chemical exfoliation of natural graphite. However, the wetting property of graphene oxide is dependent on the number of oxygen-containing groups [88]. Generally, graphene oxide displays hydrophilicity due to the chemical nature of the carboxyl and hydroxyl functional groups; therefore, proper reduction treatments must be performed to increase its hydrophobicity [88,89]. Bai and Zhang developed a reduced graphene oxide/nickel composite coating on a stainless-steel substrate via a simple electrodeposition technique. The coating was observed to possess pinecone-like micro- and nanostructures with a water contact angle of 162.7° and a sliding angle of 2.5°. Notably, the reduced graphene oxide coated surface remained remarkably superhydrophobic with excellent self-cleaning property after 100 cycles of mechanical abrasion, demonstrated by insignificant changes in the water contact angle and sliding angle to 155.8° and 5.9°, respectively [89].

CNTs are another allotropic form of carbon that can be categorized as either single-walled CNTs (SWCNTs) or multi-walled CNTs (MWCNTs). In terms of their structure and composition, CNTs are long, hollow cylindrical tubes made up of graphene sheets, whereby SWCNTs consist of a single layer of graphene with a diameter ranging from 0.4 to 2 nm, whereas MWCNTs consist of multiple layers of graphene sheets with the inner and outer diameters of 1 to 3 nm and 2 to 100 nm, respectively [90]. As CNTs are non-polar, strong, and water-resistant materials with high thermal and electrical conductivities, they are susceptible to the formation of a developed surface comprising porous agglomerates [91]. This allows CNTs to provide the essential hydrophobic features of the nanomaterial, thereby opening a wide range of opportunities for their utilization as superhydrophobic nanocoatings. A study by De Nicola et al. had fabricated a superhydrophobic MWCNT coating for stainless steel sheets via chemical vapor deposition, without the addition of any external catalysts. The MWCNT coated stainless stell exhibited a high contact angle value of 154°, indicating superhydrophobic property. This is due to the presence of low surface energy materials and the 2-fold morphology of MWCNTs that provides sufficient roughness. Collectively, this resulted in high contact angle, reduced friction, as well as drag reduction characteristics [92]. Likewise, Belsanti et al. prepared a superhydrophobic film by spraying CNT suspension on an aluminium alloy substrate. Due to the high porosity density brought upon by the presence of CNTs on the surface, the film exhibited a remarkably high contact angle of more than 160° in contrast to the bare metal [93]. In another study, Zhu et al. reported that the spraying of MWCNTs onto substrates resulted in the fabrication of a superhydrophobic coating with great reusability and easy repairability. Water droplets on the coating demonstrated a spherical shape and they could easily roll off the surface at a minimal tilt angle, owing to the low adhesive force between the water droplet and MWCNT coating. Moreover, when water droplets were released under gravity, it was observed that the water droplets can bounce away from the coated surface easily and the surface remained dry. The researchers also reported that CNT coatings that lose their superhydrophobicity can be easily regenerated due to the high thermal stability of CNTs. It was found that such superhydrophobicity can be regenerated up to five cycles, indicating the suitability of CNT-based coatings for practical applications with high abrasion [94].

#### 4.1.2. Silica-Based

Silica-based nanomaterials have been widely used in the field of superhydrophobic nanocoating. Although they are intrinsically hydrophilic, they can easily undergo chemical modifications to obtain superhydrophobicity. Excellent optical property is another advantageous feature of silica-based nanomaterials. Other favourable features of silica-based nanomaterials in the fabrication of superhydrophobic surfaces include low toxicity, mechanical and thermal stability, ease of structural regulation, as well as low preparation cost [84,95]. Wang et al. prepared a novel superhydrophobic film using triethoxyvinylsilane-modified silica nanoparticle coating on a glass substrate. The film displayed a coral-like microstructure and hierarchical roughness, which effectively repelled aqueous liquid. The superhydrophobicity of the modified silica coated surface was affirmed by a coating angle of 156°, most likely attributed to the presence of trapped air within the microstructure interspace. It was also observed that water droplets can smoothly roll across the coated surface without wetting it, suggesting effective self-cleaning property [96]. In another work, Awais et al. fabricated stearic acid modified silica nanoparticle superhydrophobic coating on a glass substrate. The study reported a maximum contact angle of 150°, indicating the superhydrophobic behaviour of the modified silica nanoparticle coating. The superhydrophobicity is thought to be conferred by the long alkyl chain of stearic acid, which is attached to the hydroxyl group of silica nanoparticles upon modification. However, the contact angle was found to drop when the drying temperature reached 260 °C, which may be attributed to the destruction of alkyl chains. Therefore, the drying temperature during the fabrication of modified silica nanocoatings must be carefully maintained to avoid the decomposition temperature of the functionalized compound [97]. Taghizadeh et al. had also developed silica nanoparticles with modified hydroxyl groups using toluene diisocyanate, followed by the grafting of alcohol chains of varying lengths. The hydroxyl groups on the alcohol further react with isocyanate groups on the silica surface, forming a urethane bond. Water droplets on the surface of modified silica nanoparticles displayed spherical shapes with a bright and visible surface underneath the water droplets, signifying the presence of trapped air. This resulted in a high apparent contact angle of 159° that is indicative of superhydrophobic behaviour. These findings can be attributed to the low surface energy and high surface roughness of silica nanoparticle coating. In addition, it was also reported that such superhydrophobicity can be retained for at least six months under atmospheric conditions, which indicates that the modified silica nanoparticles are stable for long-term applications [98]. Furthermore, Zhang et al. attempted to synthesize optically transparent superhydrophobic silica films via the sol-gel technique. The roughness of the coating was modulated by varying proportions of silica nanoparticles and silicic acid. The silica nanoparticle coated surface went through further derivatization by trimethylchlorosilane (TMCS) and hexamethyldisilazane (HMDZ) to remove any hydrophilic properties. It was reported that TMCS modified films displayed a high water contact angle of 164°, whereas HMDZ modified films displayed a water contact angle of 140°. Notably, such superhydrophobicity was also retained and almost unchanged upon abrasion and acid corrosion. However, the films lost their superhydrophobicity at temperatures above 350 °C [99]. 

In short, these findings demonstrated the feasibility of silica-based nanomaterials to be developed as novel superhydrophobic nanocoatings, as they can easily provide the ideal water contact angle for various superhydrophobic applications. Nevertheless, one of their major weaknesses is their vulnerability to thermal degradation [79]. Therefore, the determination of the strategies to improve the mechanical stability and robustness of silica-based nanomaterials will be the focus of future research. 

#### 4.1.3. Metal-Based

Metallic nanomaterials are submicron scale entities that are made up of pure metals such as gold, silver, zinc, iron, and titanium, or their compounds, such as chlorides, sulphides, oxides, and hydroxides. These nanomaterials have various advantages including high mechanical strengths, optical and magnetic properties, as well as feasibility for chemical modifications to allow better hydrophobicity. Generally, superhydrophobic nanocoatings of metallic nanomaterials are obtained through electrochemical processes [84,100]. Liang et al. prepared a nickel coated superhydrophobic film with a micro-nano binary structure via electrodeposition techniques. Upon fluorinated modification of the nickel film to obtain superhydrophobicity, it was demonstrated that the contact angle of nickel film with 5 uL water droplets was higher than 160° and had a minimum sliding angle of 1° with 10 uL water droplets. The prepared superhydrophobic film also displayed a very low contact angle hysteresis of 2.2°. Additionally, the nickel film can sustain its superhydrophobic property for more than 400 days in ambient environment, and it was stable in both strong acid and alkaline conditions. These indicated that nickel nanoparticles are durable and can be used for long-term superhydrophobic applications [101]. Another similar work by She et al. had fabricated a self-cleaning superhydrophobic surface possessing hierarchical flower-like structures via the electrodeposition of a nickel-copper nanocoating on a magnesium alloy. The coating was further modified with stearic acid to increase its superhydrophobicity. Subsequently, the surface demonstrated a high water contact angle of 167.3° with an extremely low sliding angle of approximately 1°. Stability studies indicated that the superhydrophobic surface had long-term durability and chemical stability, while pH had no influence on its superhydrophobicity [102].

In contrast to metallic superhydrophobic nanocoatings, there have been more studies involving metallic oxide nanocoatings for superhydrophobic applications. Macias-Montero et al. had deposited Ag@TiO_2_ nanorods on conventional silicon or fused silica surfaces. It was shown that the superhydrophobic behaviour of the Ag@TiO_2_ nanorod surface was of a Cassie–Baxter regime with a water contact angle of 156°, indicating that such behaviour was correlated with the presence of air pockets that are trapped within the nanorod system. However, irradiation of the surface with ultraviolet light results in a reduction in the water contact angle due to the loss of TiO_2_ superhydrophobicity, leading to complete wetting of the surface that can no longer be described by the Cassie–Baxter regime [103]. Zinc oxide nanoparticles were prepared through the sol-gel technique by Shaban et al. and were coated on cotton fabrics using spin coating methods. Such a coating demonstrated excelled superhydrophobic property with a water contact angle of 154°. The study also reported that the zinc oxide nanocoating had remarkable resistance to abrasion and had great durability under UV irradiation [104]. Another study by Hu et al. had fabricated highly stable superhydrophobic coating using a blend of TiO_2_ and SiO_2_ nanoparticles, as well as crosslinked fluorosilanes. The simultaneous use of these nanoparticles led to multi-scale roughness on the superhydrophobic surface, and at the same time, it significantly improved the hydrophobic stability of the nanocoating. This is proven by a water contact angle of 166.6° and a contact angle hysteresis of 3.4°, indicating remarkable superhydrophobicity and good self-cleaning property of the TiO_2_/SiO_2_ nanocoating [105]. Apart from that, Li et al. demonstrated excellent superhydrophobicity of a zinc oxide nanoparticle-coated surface. Here, the superhydrophobic coating was fabricated through the spraying of hydrophobized zinc oxide nanoparticles and epoxy resin. It was observed that the zinc oxide nanoparticles led to a multiscale roughness on the surface. The surface wettability of the surface was found to be influenced by the size of zinc oxide nanoparticles, in which 100 nm nanoparticles demonstrated a water contact angle and sliding angle of 150.5° and 24.5°, respectively, whereas 10 nm nanoparticles presented with a much rougher surface and higher superhydrophobicity, indicated by a water contact angle and sliding angle of 169.3° and 2.5°, respectively. The study also showed that durability can be increased by using a mixture of 10 nm and 100 nm nanoparticles, where the superhydrophobicity of such a coating is comparable to those of 10 nm nanoparticles, with a water contact angle and sliding angle of 168.5° and 3.5°, respectively [106].

### 4.2. Polymer-Based Organic Nanomaterials

Polymeric nanomaterials are regarded by researchers as the most promising materials to fabricate superhydrophobic nanocoatings due to their flexibility, processability, vast range of molecular design, as well as low production cost. Various polymeric nanomaterials such as polystyrene, polyethylene, and polypropylene have been employed to fabricate superhydrophobic surfaces, and they can produce satisfying outcomes by undergoing some simple steps of optimization. The common methods of superhydrophobic nanocoating fabrication using polymeric nanomaterials include spin coating, electrospinning, and electrodeposition [84,107]. In a study by Cheng et al., an artificial superhydrophobic surface was fabricated using high-density polyethylene on glass, copper mesh, and polyurethane sponge substrates. The researchers observed that the coated surfaces were stable in various pH conditions with remarkable thermal and wear stabilities. The high-density polyethylene coated copper mesh also displayed notable corrosion resistance and good self-cleaning property in contrast to uncoated copper mesh. All the coated substrates had demonstrated superhydrophobicity, indicated by water contact angles of more than 150°. These results proved the potential of high-density polyethylene in forming nanostructures that confer superhydrophobic properties [108]. In another work, Yuan et al. attempted to produce a lotus leaf-like superhydrophobic surface using low-density polyethylene. Through a facile synthesis technique, a low-density polyethylene superhydrophobic coating was successfully prepared with a high water contact angle and low sliding angle of 156° and 1°, respectively. The study also reported that a low drying temperature can result in increased roughness of the polymer coating, thereby maximizing the water contact angle. Thus, this work serves as the foundation for the utilization of low-density polyethylene in the development of lotus leaf-like superhydrophobic surfaces for various applications [109]. The superhydrophobic potential of polypropylene was investigated in a study by Huovinen et al., which utilized a micro-structuring technique and injection moulding. This work demonstrated a simple mass production technique for the fabrication of mechanically robust non-wetting structured polymer surfaces. The structured polypropylene surfaces presented a high contact angle higher than 150° and a low sliding angle of less than 5°, indicating that sufficient roughness for achieving the Cassie–Baxter state was present. Most importantly, the study also showed that the polypropylene nanocoating can protect fragile fine-scale surface topographies to improve durability against mechanical compression and abrasive wear [110]. Guo et al. in their study prepared a polystyrene/paraffin wax composite nanocoating on stainless steel substrate via the immersion drying technique. The maximum contact angle was 154°, attributed to the presence of microstructure on the surface roughened by polystyrene. It was also observed that the superhydrophobic surface assumed a Cassie–Baxter state where water droplets did not fill up the rough coating surface, with the presence of air in the concave structure [111].

Unlike synthetic polymeric nanomaterials, natural polymeric nanomaterials are of particular interest in the biomedical field as they comprise biologically and environmentally friendly materials. These materials are also readily and abundantly available at a much lower cost as compared to those of synthetic origin [84,112]. Chitosan is the most common example of a naturally derived polymeric nanomaterial that can be found in crustacean and insects’ exoskeletons, as well as fungi cell walls. It is a derivative of chitin composed of β,1-4 linked glucosamine and N-acetylglucosamine [112]. Wang et al. had evaluated the superhydrophobic potential of chitosan-based nanoparticle coated surfaces prepared through a one-step spray coating technique. Imaging of the chitosan coated surfaces displayed a nano- and micro-scaled roughness, resulting in remarkable superhydrophobic behaviour characterized by a water contact angle of 155°. Notably, the superhydrophobic surface remained stable for 15 days under pH conditions of 1 to 11, and a temperature of no more than 50 °C [113]. Similarly, Ivanova and Philipchenko had designed a superhydrophobic surface using chitosan-based nanoparticles. The spraying of the chitosan nanoparticle dispersion resulted in the formation of a multiscale porous textured layer that confers superhydrophobicity. Such non-wetting property was thought to be influenced by the relative number of fluoroanions per elementary unit of chitosan, whereby it was shown that a higher relative number contributed to the lowest surface charge, thus presenting the highest superhydrophobicity with a water contact angle of 156.7° [114].

### 4.3. Inorganic–Organic Hybrid Nanomaterials

The application of inorganic–organic hybrid nanomaterials as superhydrophobic nanocoating can provide a synergistic effect that combines the advantages of both organic and inorganic nanomaterials. Generally, the abovementioned carbon-based, silica-based, and metal-based inorganic nanomaterials, as well as other types of inorganic nanomaterials, can all be employed to form inorganic–organic hybrids for superhydrophobic nanocoatings with various organic polymeric nanomaterials [84]. Several studies involving inorganic–organic hybrid nanomaterials in the fabrication of superhydrophobic surfaces are discussed below.

A superhydrophobic coating was prepared in a work by Gong and He using polydimethylsiloxane (PDMS) and SiO_2_ nanoparticles via a simple spray coating method. Surface roughness was induced by the silica nanoparticles whilst PDMS was used as a low surface energy agent. As a result, the combination of the rough nanostructure with low surface energy produced an excellent superhydrophobic surface with a water contact angle and sliding angle of 156.4° and lower than 5°, respectively. Therefore, water droplets can roll off the surface easily at a slightly tilted angle. Additionally, the PDMS/SiO_2_ coating exhibited resistance towards ultraviolet irradiation, strong acid-base corrosion, as well as mechanical abrasion, indicating that the superhydrophobic surface had great mechanical stability and durability [115]. Apart from that, Pardo-Figuerez et al. in another work utilized a one-step co-continuous bilayer coating process to produce a multilayer superhydrophobic film on a polyethylene terephthalate (PET) substrate. Ultrathin polylactide fibers were first coated onto the PET films through electrospinning, which was followed by the electrospraying of nanostructured silica nanoparticles onto the coated PET/polylactide films. The results demonstrated that such a strategy of polylactide fibers and SiO_2_ nanoparticles’ co-continuous deposition had significantly increased the surface superhydrophobicity of PET substrate, characterized by a water contact angle and sliding angle of 170° and 6°, respectively [116]. One recent study by Yang et al. had synthesized a biomimetic robust superhydrophobic coating of PEG/SiO_2_/PVA/PAA/fluoropolymer possessing nano- and microstructures. It was found that the prepared surface had superior superhydrophobicity with a water contact angle of 159°, which can be attributed to the complete micro-nano rough structure due to nanoparticles aggregated with each other in a pillar-like structure. The superhydrophobic surface was also found to possess remarkable durability under mechanical shock and varying temperature conditions [117]. A zinc oxide/polystyrene superhydrophobic surface was developed using a facile and inexpensive technique in a study by Qing et al. At the zinc oxide nanoparticles to polystyrene ratio of 7:3, the contact angle was 158°, indicating superhydrophobic behaviour. Such superhydrophobicity was thought to be induced by the micro- and nano-hierarchical roughness structure of the ZnO/polystyrene nanocoating that leads to low surface energy [118]. On the other hand, CNTs are attractive nanofillers used for the reinforcement of polymeric nanomaterials due to their large surface area and aspect ratio. Chakradhar et al. had prepared superhydrophobic coatings using polyvinylidene fluoride (PVDF)-MWCNTs through spray coating. The surface had a rough, porous structure and it was reported that an increase in MWCNT content resulted in the transformation of hydrophobic state to superhydrophobic state. The highest water contact angle of 154° was observed at the PVDF to MWCNT ratio of 1:2 [119]. 

## 5. Anti-Biofilm Applications of Superhydrophobic Nanocoating

As discussed above, the process of biofilm formation typically begins with a weak, reversible adhesion of microbial cells to a surface. If these cells are not removed from surfaces, their attachment becomes stable and permanent, leading to the formation of a biofilm matrix [12,56]. Biofilms represent a high risk for chronic nosocomial infections in the clinical setting, especially in patients who received indwelling medical devices. When these medical devices are exposed to the bloodstream of patients, microbial cells in the bloodstream are provided with the opportunity to adhere to the surface of medical devices and form microcolonies that differentiate into thick, structured bacterial biofilms [120]. Moreover, medical implants are particularly susceptible to the formation of biofilms as host immune responses are usually reduced in the areas of the human body that are in contact with foreign devices. To make things worse, biofilms are mostly resistant to conventional antimicrobial therapeutics that are normally designed to target planktonic cells. This is most likely attributed to the poor penetration capability of antimicrobials into the EPS of biofilms, the presence of multidrug resistance persister cells, and the expression of resistance mechanisms by the microbial community within the biofilm [8,121,122]. Currently, the removal and replacement of the medical implant is often the only solution to biofilm-associated infections, which is very costly and traumatic to the patient, and it is not guaranteed that the recolonization of microbial cells will not occur upon the new implant [8]. Thus, it is of utmost importance to develop novel strategies that specifically target biofilm-associated infections. 

**Figure 8:**
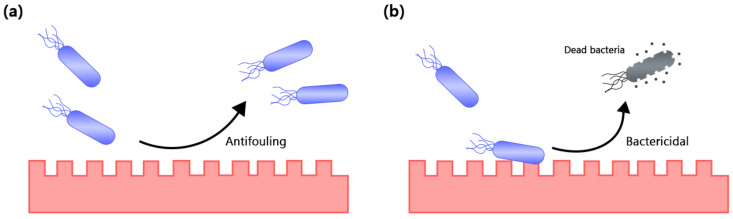
Schematic representation of the potential of superhydrophobic surfaces in preventing biofilm formation: (**a**) Antifouling property which minimizes the adhesion of bacterial cells onto the coated surface; (**b**) Bactericidal property which kills bacterial cells as a result of membrane rupture and/or the intrinsic antibacterial properties of coated nanomaterials by inducing oxidative stress, damaging biomolecules, depleting ATP, and interaction with bacterial membrane.

Superhydrophobic surfaces have been recently proposed as a potential strategy to mitigate the formation of bacterial biofilms through their antifouling properties (Figure 8a). The superhydrophobicity of a surface can be modulated by its surface charge and roughness, in which they play a significant role in determining the ability of microbial cells to adhere to surfaces of medical implants in the early stages of biofilm formation [122]. Generally, a rough surface reduces the contact area and adhesion force between microbial cells and the surface due to the presence of surface protrusions that facilitate air trapping within the surface structure as observed in the Cassie–Baxter wetting regime, thereby contributing to a high contact angle, low sliding angle, and self-cleaning property, which are the characteristic features of a superhydrophobic surface [122,123]. In addition, the local curvatures on the surface protrusions also effectively reduce the anchoring points for microbial cells [123]. One study by Wu et al. had demonstrated that micro- and nano-topographies of rough surfaces can influence the adhesion of bacteria and the subsequent formation of early biofilm. It was shown that there were significantly more bacterial cells adhered onto the electropolished smooth surfaces as compared to the untreated rough surfaces. Notably, the bacterial cells were scattered throughout the untreated rough surfaces in small clumps, whereas on the electropolished smooth surfaces, bacterial cells were present as large, clumped clusters. Hence, this study reiterated the role of rough surfaces in restraining the adhesion of bacterial cells and preventing the formation of microcolonies [124]. Another work by Crick et al. also reported that a highly rough film made from silicone elastomer had excellent superhydrophobicity of the Cassie–Baxter wetting state and had a water contact angle of 165 ± 1.31°. As compared to a flat elastomeric film, the contact between *Escherichia coli* and Methicillin-resistant *Staphylococcus aureus* to the surface was reduced, resulting in poor bacterial adhesion to the surface [125]. In terms of surface energy, it has been found that materials with a surface energy between 20 and 30 mN/m can produce better antifouling effect. Moreover, it has been shown that fluorinated nanocoatings can effectively prevent fouling on surfaces as they can modulate surface free energy to be approximately 20 to 30 mN/m [126,127]. This corresponds to the findings in a study by Yuan et al., which demonstrated that the lowering of polystyrene surface energy by fluorination significantly lowered the adherence of *Escherichia coli.* The surface also displayed a self-cleaning ability with a water contact angle of more than 150°, indicating that the initially adhered bacterial cells can be easily removed from the superhydrophobic surface by washing [127]. 

Apart from that, micro- and nano-textured surfaces brought upon by superhydrophobic nanocoating can also provide bactericidal effect in addition to their antifouling properties (Figure 8b). It was thought that such bactericidal property is due to the rupturing of bacterial cell membrane when the cells are stretched and suspended between the interspacing of micro- and nanostructures of superhydrophobic surfaces. However, such an effect is more prominent in Gram-negative bacteria due to the presence of fewer peptidoglycans layers that conferred higher physical resistance as compared to Gram-positive bacteria [128]. Jenkins et al. in their study developed titanium dioxide nanopillars coated on a titanium substrate. It was reported that the nanostructured surface induced envelope deformation and penetration of bacteria, thereby reducing the capacity of bacteria to replicate on such surfaces and enhanced the anti-biofilm property [129]. 

In addition, certain nanomaterials have been reported to possess antibacterial properties, such as those of amphoteric metal oxides and silver nanoparticles which impart bactericidal activity via oxidative stress induction, the release of metal ions, biomolecule damage, the depletion of ATP, and membrane interaction [130,131]. These nanomaterials can be incorporated with other nanomaterials during the fabrication of superhydrophobic nanocoatings to provide a synergistic effect in preventing the formation of biofilms. As such, the adhesion of both Gram-positive and Gram-negative bacteria can be prevented whilst significantly inhibiting the growth of bacterial cells that managed to adhere on surfaces, thus no subsequent biofilm formation is possible [130,132]. Liu et al. fabricated a fluorinated silver nanoparticle surface with a nano-porous structure and evaluated its antibacterial properties. The fabricated surface demonstrated superhydrophobicity with a water contact angle of 163° and a contact angle hysteresis of 1°, where water droplets were observed to float on the coated surface. It was also found that the coated surface significantly inhibited the growth of Gram-negative bacterial strains and presented antifouling properties to the substrate [133]. Likewise, Spasova et al. developed a superhydrophobic nanocoating using poly(vinylidene fluoride) and poly(vinylidene fluoride-co-hexafluoropropylene) with zinc oxide nanoparticles. The coating displayed superhydrophobicity with a contact angle of 152° and had antimicrobial effects against *Escherichia coli* and *Staphylococcus aureus*. Due to the superhydrophobic nature of the coated surface, the adherence of bacterial cells was greatly reduced, suggesting antifouling properties that affect the initial attachment phase of biofilm formation. Such antimicrobial effect was also reported to be attributed to the presence of ZnO nanoparticles that conferred bactericidal properties, killing the small number of bacterial cells that adhered to the coated surface [134]. In a nutshell, these studies suggested that superhydrophobic surfaces can impart anti-biofilm behaviour to nanocoated surfaces through their antifouling and bactericidal properties as a result of high surface roughness, low surface energy, as well as the presence of nano- and micro-structures. 

**Figure 9:**
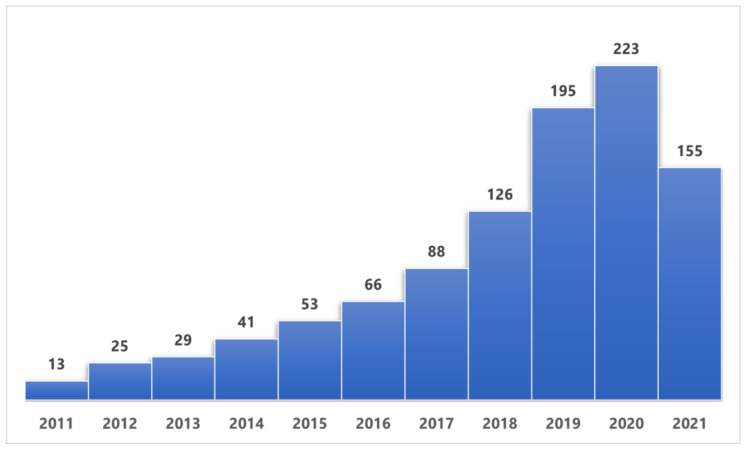
Number of yearly studies conducted on the effects of superhydrophobic nanocoatings in preventing biofilm formation from 2011 to 2021. Data are obtained from a simple search of published research literatures in Google Scholar database using the keywords “superhydrophobic”, “anti-biofilm”, “antibacterial”, and “antimicrobial”.

Apart from those discussed above, there are multiple other studies conducted over the years that evaluated the potential of superhydrophobic nanocoatings on surfaces to mitigate the formation of bacterial biofilms (Figure 9). We have summarized some of the notable studies performed in this field of research and highlighted the key findings from these studies in Table 4. 

The promising results obtained from various studies on the potential of superhydrophobic surfaces in preventing biofilm formation have prompted researchers to further investigate the feasibility of such surfaces when used on indwelling medical devices. Zhang et al. synthesized a multi-layered superhydrophobic coating for urinary catheters. In the study, polydopamine nanocoating was utilized as the platform for anchoring silver nanoparticles, followed by hydrophobic modification of the nanocoating using 1H,1H,2H,2H-perfluorodecanethiol. The surface displayed a micro- and nano-hierarchical structure with high water repellence, indicated by the water contact angle of 154.7 ± 1.1° and sliding angle of less than 5°. The superhydrophobic catheter showed remarkable antibiofilm properties against *Escherichia coli* and *Proteus mirabilis*. When compared with the commercial all-silicone and silver-alloy-hydrogel catheters, the superhydrophobic nanocoated catheter reduced bacterial migration and biomass accumulation by up to 55% and 90%, respectively. The enhanced anti-biofilm behaviour was the synergistic effect of superhydrophobicity and the intrinsic antibacterial activity of silver nanoparticles [154]. In another recent work, Chae et al. had modified the surface of an orthopaedic implant by coating its surface with a self-assembled monolayer of 1H,1H,2H,2H-perfluorooctyltriethoxysilane. The coating was found to significantly lower the surface energy of the micro- and nano-hierarchical structured surface, thereby enhancing its superhydrophobicity. Remarkably, the adherence of both *Pseudomonas aeruginosa* and Methicillin-resistant *Staphylococcus aureus* was not observed in both the short and long term, indicating the excellent antifouling property and mechanical durability of the coated orthopaedic implant. These findings were attributed to the trapped air pockets within the hierarchical structure that effectively minimized contact between the bacterial suspension and the surface [155].

Despite these studies that presented the anti-biofilm potential of superhydrophobic surfaces, there have been several contradicting reports on the anti-biofilm efficacy of such surfaces (Table 5). This may be attributed to the fact that rougher surfaces possess greater surface area due to the presence of multiple pits and grooves, thereby leading to higher bacterial adherence as they have more favourable sites for colonization [156]. For instance, Sousa et al. had reported that *Staphylococcus aureus* and *Pseudomonas aeruginosa* demonstrated significantly higher colonization and greater adherence on a rough superhydrophobic poly(L-lactic acid) surface in contrast to a smooth surface, leading to biofilm formation [157]. Such conflicting conclusions with regard to the effect of surface roughness on bacterial adherence may be potentially due to the dependency of surface wettability on the interactions between hydrophobicity and roughness [158]. Indeed, superhydrophobic surfaces can limit bacterial adherence due to air being trapped within the surface structures. Nevertheless, Marmur has reported that multiscale roughness does not represent an indispensable requirement in achieving superhydrophobicity, as it is often achieved with the combination of surface structure at the micro- and nanometre scale [159,160]. It is also important to note that surfaces with drastically varying topography may possess similar roughness parameters, as roughness is only the description of the height variation of a surface, while topography describes the configuration of a surface in a three-dimensional space. Thus, studies are currently focused on fabricating superhydrophobic surfaces with precisely defined topography instead of merely depending on roughness measurements [158,159,160]. A study by Encinas et al. had demonstrated that surface microstructures of 0.2 to 1 µm, which fall just below the typical size of bacterial cells, had the advantage of preventing the fitting of bacterial cells within the sufficiently small spacing between the local curvatures. At the same time, the spacing is sufficiently large to introduce superhydrophobicity with a contact angle of 176 ± 3°, which effectively reduces adhesion points for bacterial cells. This proved that the three-dimensional topography of a surface is crucial in determining the antifouling properties of a superhydrophobic surface [123]. However, the detailed optimal topography of a superhydrophobic surface that can produce superior anti-biofilm property is yet to be fully elucidated to-date, and works are still ongoing to determine the exact correlation between surface roughness, topography, and bacterial adherence [159,160]. On the other hand, the process of biofilm formation is also highly dependent on factors such as the regulation of QS and other cell-to-cell communication processes, instead of solely dependent on the initial physical binding of bacterial cells to surfaces [161]. A study by Ellinas et al. had also reported the existence of a bacterial concentration threshold, in which the antibacterial action of any superhydrophobic surface will be compromised above such threshold. The results showed that the antibacterial action of a superhydrophobic micro- and nanotextured poly(methyl methacrylate) surface faded when the surface bacterial density exceeded 6.7 × 10^8^ cfu/cm^2^. This phenomenon is observed when the antiadhesive properties of a superhydrophobic surface are insufficient to halt bacterial growth, leading to the formation of biofilm [162]. Although a hybrid superhydrophobic surface that comprises both antiadhesive and bactericidal properties may be a feasible approach, further studies that elucidate the exact roles of these surfaces in preventing biofilm formation shall be of particular research focus in the coming years.

## 6. Conclusions and Future Perspectives

Bacterial biofilms are a major public health issue as they have been widely recognized to play a role in the pathogenesis of various chronic polymicrobial infections. Specifically, the formation of bacterial biofilms on indwelling medical devices that leads to device-associated infections is the most common and feared complication in the clinical setting. Under the protection of a self-produced extracellular polymeric substance, microbial cells within the biofilm can become resistant and tolerant to host immune responses and antimicrobial therapeutics, which makes the treatment of device-associated nosocomial infections notoriously challenging for healthcare professionals. The decreased susceptibility of biofilm to antimicrobial therapeutics can also be attributed to the differences in structural and genetic features, microenvironment, as well as growth rate with those of planktonic microorganisms. Thus, the design of novel strategies to manage these infections should be expedited to provide an alternative to existing antimicrobials and device replacement surgeries. Among the various approaches that have been studied thus far, superhydrophobic nanocoating is believed to be a promising strategy in preventing the formation of bacterial biofilms by altering the physical properties of biomaterials used in indwelling medical devices. Superhydrophobicity is generally described by a surface that possesses a high water contact angle of more than 150°, high surface roughness, low surface energy, and low contact angle hysteresis. Inspired by nature, superhydrophobicity can be fabricated on surfaces by using a vast range of inorganic and organic nanomaterials via various coating techniques. In terms of their potential in preventing device-associated infections, superhydrophobic surfaces can reduce the adhesion of microbial cells through their antifouling properties. Such antifouling properties are the result of protrusions and a rough topography on superhydrophobic surfaces. In addition, superhydrophobic surfaces can also impart bactericidal activity by rupturing the cell membrane of microbial cells when they are suspended and stretched within the interspacing present between the micro- and nanostructures of the surface. As the initial attachment of microbial cells to a surface is regarded as the most crucial step in initiating the processes leading to biofilm formation, these features of superhydrophobic surfaces can effectively prevent the transformation of microbial cells from their planktonic state into biofilms. In addition, certain nanomaterials can provide intrinsic bactericidal activity in addition to those of the superhydrophobic surface, such as those of metallic nanoparticles, thereby resulting in a synergistic anti-biofilm behaviour. Over the years, advancements in the field of nanotechnology have led to the development of various nanomaterials, whereby multiple studies have indicated that superhydrophobic nanocoatings fabricated from these nanomaterials have the potential to be developed as a promising solution for various biofilm-associated nosocomial infections. However, most of the studies discussed herein are performed on non-medical device substrates. Therefore, further studies must be conducted to evaluate the feasibility of superhydrophobic nanocoatings when used directly on indwelling medical devices. Nonetheless, it has been recently reported that the disruption of the initial adherence of microbial cells did not offer significant impact on the overall occurrence of biofilm formation, as the process is highly dependent on the regulation of quorum sensing mechanisms instead of the amount of microbial cell adherence. Hence, more in-depth studies are required to elucidate the exact role and mechanisms of superhydrophobic surfaces in mitigating biofilm formation in order to pave the way for successful clinical translation in the near future.

## Figures and Tables

**Table 1:** Examples of medical device-associated bacterial infections and their common causative pathogens.

Type of Medical Device-Associated Bacterial Infections	Common Causative Pathogens	Reference(s)
Central line-associated bloodstream infection	Coagulase-negative *Staphylococci* *Staphylococcus aureus* *Enterococcus* spp. *Pseudomonas* spp.	[11,13]
Catheter-associated urinary tract infection	*Escherichia coli* *Pseudomonas* spp. *Enterococcus* spp. *Staphylococcus aureus* Coagulase-negative *Staphylococci* *Enterobacter* spp.	[12,17]
Ventilator-associated pneumonia	*Pseudomonas* spp. *Klebsiella* spp. *Enterococcus* spp. *Staphylococcus aureus* *Pseudomonas aeruginosa* *Acinetobacter baumannii*	[11,17]
Prosthetic heart valve infection	*Staphylococcus aureus* *Staphylococcus epidermidis* *Streptococcus* spp.	[11]
Surgical site infection	*Staphylococcus aureus* *Enterococcus* spp. *Acinetobacter* spp. *Pseudomonas* spp. *Escherichia coli*	[17]

**Table 2:** Summary of the functions of exopolysaccharides and their relevance to biofilms.

Function	Functional Relevance to Biofilms	Reference(s)
Adhesion	Facilitates the initial steps of bacterial colonization on surfaces. Allows long-term attachment of bacterial cells.	[14,22]
Bacterial aggregation	Enables bridging between bacterial cells and cell-to-cell recognition. Allows immobilization of bacterial population, resulting in high cell densities on biofilms.	[14,21,22]
Retention of water	High water retention of hydrophilic and charged exopolysaccharides allows the maintenance of hydrated microenvironment. Allows the survival of desiccation in water-deficient states.	[14,21,22]
Cohesion	Mediates the mechanical stability of biofilms in association with multivalent cations. Determinant of biofilm architecture. Allows cell-to-cell communication.	[14,22]
Protective barrier	Confers resistance to both specific and non-specific host immune responses during infection. Confers tolerance towards various antimicrobial and disinfectant treatments.	[21,22]
Source of nutrients	Serves as source of phosphorus, nitrogen, and carbon containing compounds that can be utilized by the bacterial population. Mediates the sorption and accumulation of nutrients from surrounding environment.	[14,22]
Binding of enzymes	Interacts with non-glycolytic extracellular enzymes leading to increased retention, stabilization, and accumulation of bacterial cells.	[22]

**Table 3:** Examples of naturally occurring superhydrophobic surfaces.

Natural Superhydrophobic Surface	Water Contact Angle	Reference(s)
*Nelumbo nucifera* (Lotus leaf)	160°	[73,74]
*Oryza sativa* L. (Rice leaf)	157°	[75,76]
*Colocasia esculenta* (Taro plant leaf)	164°	[74,76]
*Setcreasea purpurea* boom (Purple setcreasea)	167°	[75,76]
*Polygonum perroliatum* L. (Perfoliate knotweed)	162°	[75,76]
*Gerris remiges* (Water striders)	167.6°	[77]
*Meimuna remiges* (Homoptera)	165°	[75,76]
Diptera *Tabanus chrysurus*	156°	[75,76]
Cicada wings	152°	[75]

**Table 4:** Prior works that demonstrated notable anti-biofilm potential upon superhydrophobic nanocoating on substrate.

Substrate	Coated Nanomaterial (s)	Surface Pattern Scale	Water Contact Angle	Findings	Reference
Stainless steel	Silver nanoparticles treated with fluorosilane	Micro and nano (~200 nm)	154°	Micro-structured superhydrophobic surface was observed. Reduced bacterial adhesion by 88%. Efficient against both Gram-positive and Gram-negative microorganisms.	[135]
Titanium	Titanium dioxide nanotubes treated with perfluorooctyl-triethoxysilane	Nano (~400 nm)	156°	Inhibited the adherence of *Staphylococcus aureus* on the surface. Anti-biofilm effect attributed to the roughened surface and lowered surface energy.	[136]
Stainless steel	Fluorosilane modified polystyrene/Ag microspheres	Nano (~40 nm)	157.1°	Enhanced bacterial anti-adhesive properties of 98.3% and 99.4% against *Staphylococcus aureus* and *Escherichia coli*, respectively. Outstanding superhydrophobicity and self-cleaning properties of a Cassie–Baxter wetting regime.	[137]
Stainless steel	MWCNTs	Micro and nano (5–15 µm)	153.82 ± 1.19°	90% average reduction in biofilm formation of *Escherichia coli* and *Staphylococcus aureus*. Self-cleaning properties indicated by efficient sliding of bacterial cells from the surface without leaving any noticeable debris.	[138]
Aluminum	Silica nanoparticles modified with fluorosilane	Nano (~200 nm)	159 ± 1°	Coated surface produced 6.5 ± 0.1 and 4.0 ± 0.1 log-cycle reductions in bacterial surface colonization by *Salmonella* Typhimurium LT2 and *Listeria innocua*, respectively. Effect attributed to presence of interstitial air on the surface which reduces surface contact area.	[139]
Denture base resin	Hydroxyl functionalized fluoropolymer, polyurethane oligomer, epoxy group functionalized SiO_2_ nanoparticles	Micro and nano (189.6 nm)	155.9°	Presence of air layer in the void between the surface roughness indicated Cassie–Baxter wetting state. Decreased contact area between bacterial cells and the surface, thereby reducing adherence. Significant suppression of bacterial colonization on surface.	[140]
Thermoplastic polyurethane sheets	Polydimethylsiloxane and silver phosphate nanoparticles	Nano (Length not specified)	152°	Increased surface roughness and packed nanostructures obtained at higher nanoparticle concentration. 80% reduction in the adhesion of *Staphylococcus aureus* and *Escherichia coli* to the coated surface. Anti-adherence effect attributed to reduced solid–liquid interactions and mechanical rupture of bacterial membrane by surface nanostructures.	[141]
Aluminum foil	Polyfurfuryl alcohol, fluorinated acrylic copolymer and silica nanoparticles	Nano (20–40 nm)	>150°	Biofouling resistance demonstrated by low bacterial adhesion of *Escherichia coli*, *Staphylococcus aureus*, and *Pseudomonas aeruginosa* to the coated surface.	[142]
Glass	Polydimethylsiloxane and copper nanoparticles	Micro and nano(3–5 µm)	151 ± 2°	Surface coating prevented the adhesion of *Staphylococcus aureus* and *Escherichia coli.* A 4-log reduction in the numbers of bacterial cells was observed.	[143]
Etched filter paper	Cellulose nanofibers with titania-perfluorooctyl trimethoxysilane	Nano(Length not specified)	158°	Rough morphology inhibited the adhesion of *Escherichia coli*. Surface curvatures and anti-wetting effects synergistically inhibited bacterial deposition.	[144]
Glass	Fluorinated silica colloids	Micro and nano (Length not specified)	167.7 ± 1.8°	Reduced the adhesion of *Staphylococcus aureus* and *Pseudomonas aeruginosa* by 2.08 ± 0.25 and 1.76 ± 0.12, respectively. Antifouling effect attributed to high surface roughness and low surface energy from fluorosilane modification.	[145]
Copper alloy	Copper nanoparticles with perfluorooxysilane	Micro and nano (5–10 µm)	170.1 ± 1.5°	Both *Escherichia coli* and *Klebsiella pneumoniae* were pierced and deformed with damaged cellular membranes due to contact with the hierarchically roughened surface. Impeded the primary adhesion of bacterial cells due to electrostatic and steric repulsions. Bacterial cells damaged by copper ions due to generation of reactive oxygen species and DNA degradation.	[146]
Copper alloy	Copper nanoparticles with fluorooxysilanes	Micro and nano (Length not specified)	171°	Reduced the contact area for bacterial cell deposition. Sustained release of copper ions led to bactericidal activity towards *Escherichia coli*.	[147]
Titanium alloy	Silanized titania nanoflower	Nano(823.6 ± 163.6 nm)	156.4 ± 3.8°	Significantly reduced the adhesion of both Gram-negative and Gram-positive microorganisms on the surface. Cassie–Baxter regime was achieved from the combination of surface roughness, nanotopography of titania nanoflower, and presence of low surface energy, leading to minimized bacterial contact area.	[148]
Copper foil	Copper (I) oxide nanopetals	Nano (200–400 nm)	154 ± 0.6°	Effectively halted the adhesion of bacterial cells with no biofouling activity. Exhibited bactericidal property attributed to the release of copper ions. Higher bactericidal activity observed in *Escherichia coli* as compared to *Bacillus subtilis* due to the simplistic cell wall structure of Gram-negative microorganisms.	[149]
Glass	Fluorinated silica/copper (II) oxide nanoparticles	Micro and nano(Length not specified)	160°	The coated surface was highly resistant to bacterial adhesion, demonstrated by 3.2 log reduction in *Escherichia coli*. Water-repellent property due to micro- and nano-scale structure and low surface energy. Excellent bactericidal performance due to release of copper ions.	[150]
Polyurethane sponge	Zinc oxide/copper nanoparticles and perfluorooctyltriethoxysilane	Micro and nano (Length not specified)	161.6 ± 1°	Significantly reduced the adhesion of *Staphylococcus aureus* by up to 99.9% over a four-day period. Low surface energy effectively prevented the adhesion of bacterial cells.	[151]
Copper	Silver nanoparticles with fluorosilane	Micro and nano (~300 nm)	152°	Effectively inhibited the adhesion of bacterial cells due to low surface energy. Silver nanoparticles generated a sustained flux of silver ions that damaged the bacterial cells.	[152]
Polymer films	Poly(L-lactide) and modified silica nanoparticles	Micro and nano (1–2 µm)	157°	Significantly reduced the adhesion of *Staphylococcus aureus* on the surface. Anti-adhesive property attributed to the presence of trapped air in the microstructures, which reduced surface contact with bacteria.	[153]

**Table 5:** Examples of contradicting findings on the anti-biofilm potential of superhydrophobic surfaces.

Substrate	Coated Nanomaterial(s)	Water Contact Angle	Findings	Reference
Glass	Poly(L-lactic)-dioxane	154°	Superhydrophobic surface supported a greater amount of *Staphylococcus aureus* cells and enabled the formation of *Pseudomonas aeruginosa* biofilms. Less bacterial removal was observed as compared to the smooth surface.	[157]
Glass	Trimethylmethoxysilane	>150°	Surface superhydrophobicity enhanced the adhesion of *Escherichia coli* and *Bacillus subtilis*. High adhesion attributed to large surface contact area from high surface roughness.	[163]
Titanium	-	166 ± 4°	Colonization of *Staphylococcus aureus* was observed on the superhydrophobic surface.	[164]
